# Graft-versus-cancereffect and innovative approaches in the treatment of refractorysolid tumors

**DOI:** 10.3906/sag-1911-112

**Published:** 2020-11-03

**Authors:** Uğur ŞAHİN, Taner DEMİRER

**Affiliations:** 1 Hematology Unit, Yenimahalle Education and Research Hospital, Yıldırım Beyazıt University, Ankara Turkey; 2 Department of Hematology, School of Medicine, Ankara University, Ankara Turkey

**Keywords:** Graft-versus-tumor effect, recipient derived immune effector cells, allogeneic hematopoietic stem cell transplantation, solid tumors

## Abstract

**Background/aim:**

Allogeneic hematopoietic stem cell transplantation (allo-HSCT) has been used for the treatment of various refractory solid tumors during the last two decades. After the demonstration of graft-versus-leukemia (GvL) effect in a leukemic murine model following allo-HSCT from other strains of mice, graft-versus-tumor (GvT) effect in a solid tumor after allo-HSCT has also been reported in a murine model in 1984. Several trials have reported the presence of a GvT effect in patients with various refractory solid tumors, including renal, ovarian and colon cancers, as well as soft tissue sarcomas [1]. The growing data on haploidentical transplants also indicate GvT effect in some pediatric refractory solid tumors. Novel immunotherapy-based treatment modalities aim at inducing an allo-reactivity against the metastatic solid tumor via a GvT effect. Recipient derived immune effector cells (RDICs) in the antitumor reactivity following allo-HSCT have also been considered as an emerging therapy for advanced refractory solid tumors.

**Conclusion:**

This review summarizes the background, rationale, and clinical results of immune-based strategies using GvT effect for the treatment of various metastatic and refractory solid tumors, as well as innovative approaches such as haploidentical HSCT, CAR-T cell therapies and tumor infiltrating lymphocytes (TIL).

## 1. Introduction

High dose chemotherapy with autologous hematopoietic stem cell transplantation (HSCT) could not achieve the expected treatment success in patients with solid tumors [u2901, u2904]. Allo-HSCT has been used for the treatment of various refractory solid tumors during the last two decades. GvT effect in a solid tumor after allo-HSCT has also been reported in a murine model in 1984 after the demonstration of graft-versus-leukemia (GvL) effect in a leukemic murine model following allo-HSCT from other strains of mice [u2905]. Phase I and II trials using allo-HSCT with RIC conducted by the European Society for Blood and Marrow Transplantation Solid Tumors Working Party (EMBT-STWP) have reported the presence of a GvT effect in patients with various refractorysolid tumors,including renal, ovarian and colon cancers,as well as soft tissue sarcomas[1].The growing data on haploidentical transplants also indicate GvT effect in some pediatric refractory solid tumors [21–25].

The standard chemotherapy-based approaches have been shifting towards immunotherapy-based modalities, which aim at inducing an allo-reactivity against the metastatic solid tumor via a GvT effect [13, 26–33]. The acceptable toxicity profile has enabled allo-HSCT with RIC to be an alternative for the elderly and medically fragile patients with refractory metastatic solid tumors[u2907].The evolving evidence has also indicated the potential role of recipient derived immune effector cells (RDICs) in the antitumor reactivity following allo-HSCT, which has been considered as an emerging therapy for advanced refractory solid tumors[u2908].

This review summarizes the background, rationale, and clinical results of immune-based strategies using GvT effect for the treatment of various metastatic and refractory solid tumors, as well as innovative approaches such as haploidentical HSCT, CAR-T cell therapies and tumor infiltrating lymphocytes (TIL).

## 2. Cytotoxic adoptive T-cell therapy

Novel approaches including adoptive T-cell therapy (ATCT), targeted therapies and allo-HSCT with RIC are able to induce more durable responses via the advantage of a GvT effect [u2909]. Better understanding the mechanisms behind the adoptive immune system has enabled the establishment of new targets for the treatment of various solid tumors [35]. The GvT effect and tumor response after allo-HSCT with RIC depend on the activity and interaction of RDICs, leukocyte-activated killer cells (LAKs) and cytokine-induced killer cells (CIKs). Thus, it may also be regarded as a nonspecific ATCT. ATCT involves the expansion of cytotoxic immune effector cells of either donor or recipient type [36]. According to results of some early phase trials, ATCT may be a potent immunotherapeutic approach in refractory solid tumors [35]. There remains much more to be discovered regarding the interactions of T-cell subsets, mechanisms of GvT effect and differences between GvL effect of hematologic malignancies and GvT effect in refractory solid tumors.

## 3. Graft-versus-tumor effect

Graft-versus-host disease (GvHD) and therefore GvL effect occurring after allo-HSCT contributes to and maintains an antileukemic feature [37, 38]. Chronic GvHD generally leads to a more potent GvL effect than acute GvHD [39]. The duration of remission is reported to be higheramong patients with GvHD when compared to ones without GvHD [40]. Indirect evidences for the presence of an immune-mediated GvL effect include the lower risk of relapse among patients undergoing allo-HSCT when compared to autologous HSCT and an increased risk of relapse among patients receiving T-cell depleted transplants [41, 42]. The direct evidence of GvL effect can be interpreted from the posttransplant studies reporting an augmentation of GvL effect following donor lymphocyte infusions (DLI) after allo-HSCT [43]. DLI without cytotoxic therapyis associated with a high rate and durability of remissionwhen used for the treatment of relapse after allo-HSCT [44–46].

The activation of Fas-dependent killing and perforin degranulationvia the GvL effect, which is mediated by donor T-cells (CD4+, CD8+ and natural killer – NK-cells), eradicates malignant cells [47, 48]. Interferon-C, interleukin-2 and tumor necrosis factor-αare the main cytokines that potentiate the GvL effect [49]. Posttransplant ATCT against human cancer-associated antigens, T-cell receptor genes or minor histocompatibility antigens (e.g.; HA-1,HA-3, etc.) may also induce antitumor effects [50].

The development of acute and chronic GvHD after allo-HSCT, which is an immuno-modulatory therapy aiming at exploiting a GvT effect for solid tumors, has been linked to a better response rate[1]. The identification of antigen targets of donor and RDICs and development of targeted therapies may further increase the GvT effect of allo-HSCT for solid tumorsandalso reduce the treatment toxicity[1]. However, the critical balance between effective immunosuppression, GvHD and relapse still remains as amajor concern.

### 3.1. GvT effect in renal cell carcinoma

Although RCC is sensitive to immunotherapy, interferon-α with or without interleukin-2 (IL-2) yields unsatisfactory response (10%–20%) and long-term progression-free survival (PFS) rates of 4%–15% [51–53].Although the introduction of novel immunotherapeutic agents, such as anti-PDL-1 antibodies (nivolumab and ipilimumab) provided some improvement in overall survival rates of RCC patients, none of the current drugs have a curative potential in RCC [54].

Allo-HSCT with RIC has been considered as a promising option on the basis of GvT effect in this setting [27, 28, u2913, u2914]. The first series of allo-HSCT with RIC reported a 53% response rate for cytokine-refractory RCC[27].In the largest series of allo-HSCT with RIC in RCC patients by the EBMT-STWP, in which a fludarabine-based conditioning was administered to all 124 patients, TRM at the end of first year was 16% and mostly associated with acute GvHD [56]. A complete response was achieved in 4 patients at a median of 150 (42–600) days posttransplant with an overall response rate of 22.5%. Another trial with 75 metastatic RCC patients receiving allo-HSCT with RIC reported a sustained engraftment in 74 out of 75 patients [57]. The frequency of chronic GvHD was 50% and associated with a significant tumor response.

As a result, a reasonable GvT effect in RCC patients receiving allo-HSCT with RIC was documented especially in the presence of chronic GvHD, which led to an increase in survival rates.

### 3.2. GvT effect in refractory and resistant colorectal cancer

The median survival in refractory and resistant colon cancer still remains as low as 9 to 12 months after second-line treatment [58]. The addition of monoclonal antibodies, such as cetuximab or bevacizumabto combination chemotherapiesmay partially increase remission and survival rates. However, durable remission usually cannot be achieved, especially in the presence of resistant disease [59, 60]. Allo-HSCT with RIC has been studied as animmunotherapy-based therapeutic strategy for the management of metastatic colorectal cancer (mCRC)[u2916, u2917]. Hentsschke et al. reported 6 mCRC patients receiving allo-HSCT with RIC, which yielded 1 complete response and 1 mixed response [62]. In amulticenter trial by EBMT, 39 patients with mCRC had allo-HSCT with RIC and all patients engrafted (mediandonor T-cellchimerism of 90% at day +60). Transplant-related morbidities were limited. Grades II-IV acute GvHD occurred in 14 patients (35%) and chronic GvHD in 9 (23%). TRM occurred in 4 patients (10%). The best tumor responses were: 1 complete response (CR) (2%), 7 partial response (PR) (18 %) and 10 stable disease (SD) (26%), leading to an over all disease control in 18 of 39 patients (46%)[63,64]. The exploitation of GvT effect with allo-HSCT in refractory mCRC might be an alternative to conventional strategies and may sometimesyield favorable outcomes, especially in the presence of chronic GvHD, among young patients with refractory mCRC.

### 3.3. GvT effect in refractory ovarian cancer

Bay et al. reported 5 refractory ovarian cancer (OC) patients receiving allo-HSCT with RIC. Tumor regression were observed in 4 patients during acute or chronic GvHD and relapse occurred in 1 patient treated with methylprednisolone for chronic GvHD [65]. EBMT-STWP also evaluated 17 heavily pretreated refractory OC patients, retrospectively. Mortality was reported in 11 patients, 8 of whom died of tumor progression at a median follow-up of 296 days (range 51–599) [66]. Grades 2–4 acute GvHD was seen in 8 patients, 7 (41%) of whom had a partial response. DLI was associated with a tumor regression in 1 out of 3 patients. These data support the presence of a GvT effect associated with the severity of GvHD. Another retrospective multicenter study including 30 OC patients receiving allografts reported that the presence of chronic GvHD was associated with a significantly higher overall survival (OS) rate (17.6 months vs. 6.5 months, P < 0.05).An objective response rate of 50% and TRM of 20% were reported at the end of first year [67]. Median OS was 10.4 months with a median follow-up of 74.5 months (range 16–148 months).

### 3.4. GvT effect in breast cancer

Morecki et al. demonstrated a GvT effect in mice implanted with 4T1 mammary carcinoma cell line and given minor histocompatibility mismatched DBA/2 spleen cells [68]. This direct GvT effect mediated by the alloreactive donor splenocytes in the absence of any anticarcinoma agents has also been demonstrated by direct inhibition of liver metastases through intraportal inoculation of allogeneic splenocytes, but not syngeneic splenocytes [69].

The first report of allo-HSCT in metastatic breast cancer (BC) was published by Eibl et al. in 1996 [13]. The advantages of allo-HSCT over autologous HSCT for metastatic BC are i) cancer-free graft and ii) immune-mediated GvT effects mediated by human leukocyte antigen compatible donor T-cells [1, 33, u2919].After the demonstration of tumor regression in metastatic BC via allogeneic T-cell mediated GvT effects in several murine models [u291a], a study by the National Cancer Institute including 16 metastatic BC patients investigated whether a clinical graft-versus-BC effect existed via allogeneic lymphocytes after allo-HSCT from HLA-matched siblings following a RIC regimen. In order to avoid the overlap of immunological GvT effect and antitumor effect of cytotoxic chemotherapy used in the pretransplant conditioning regimen, allogeneic T-lymphocytes were removed from the stem cell graft and were subsequently administered at escalating doses after allo-HSCT (on +42, +70, and +98 days). Objective tumor regression occurred in 6 patients 28 days after allo-HSCT. Disease progression following allo-HSCT was observed before subsequent tumor regression in 2 patients. Tumor regressions obtained simultaneously with the accomplishment of complete donor T-lymphoid engraftment were associated with the development of GvHD and abrogated after systemic immunosuppression[u291b].

### 3.5. GvT effect in soft tissue sarcomas

Immune-mediated effect against soft tissue sarcomas (STS) has been shown in experimental animal models of allo-HSCT [u291c]. Most of the evidence comes from case reports and small series of patients transplanted from HLA-matched siblings. Despite several reports ofthe presence of a graft-versus-sarcoma effect, [u291d] tumor regression following allo-HSCT with RIC regimens has not been reported among patients with various histologic subtypes [76]. A retrospective study by Secondino et al. evaluated 14 adult patients with advanced STS receiving allo-HSCT with RIC in the EBMT database. Overall, acute GvHD was reported in 9 patients (64%). Grades 3–4 acute GvHD was observed in 4 (28%) and grade 2 in 5 cases (36%). Chronic GvHD occurred in 4 out of 9 evaluable patients (44%) and was extensive in 2. Four patients experienced durable disease stabilization following allo-HSCT [77]. A well designed phase 2 study, enrolling patients with limited tumor burden and slow growing tumors, may help to define the possible role of allo-HSCT with RIC in patients with STS in whom conventional treatments have failed.

### 3.6.GvT effect of haploidentical stem cell transplantation in refractory solid tumors

Innovative allo-HSCT approaches such as haploidentical HSCT, which takes advantage of GvT effects in order to control disease, while minimizing the treatment related mortality or scale of GvHD, are being studied in many recent clinical trials [21–24]. The evidence of haploidentical HSCT in solid tumors are mainly limited to pediatric solid tumors such as neuroblastoma and sarcomas [21–23]. A pilot study by Lang et al. evaluated the feasibility and toxicity of transplantation of haploidentical T and B-cell depleted grafts with high numbers of NK cells. Since grade 2 acute GvHD was observed in 4 patients andchronic GvHD in 2, it was concluded that haploidentical HSCT is feasible with low toxicity even in intensively pretreated patients with neuroblastomas and sarcomas [21]. Llosa et al. also reported the results of haploidentical stem cell transplantation with RIC in 16 pediatric and adolescent, as well as young adult patients with solid tumors. A limited GvHD was seen in 3 patients and non-relapse mortality in 1 patient. This approach may serve as a platform for posttransplant strategies to prevent relapse and optimize PFS[22].

## 4. The role of recipient derived immune effector cells in the antitumor effects

The anticancer effect of RDICs was first time suggested by Alexander et al. in 1996. They reported that xenogeneic lymphocytes from tumor immunized sheep reduced fibrosarcoma growth in immuno competent rats. The observed anticancer effect was not mediated via direct antitumor activity of donor T-cells as these were rapidly rejected in the xenogeneic setting, rather a “messengersignal” created by the infused xenogeneic donor cells in directly boosted recipient’s immune reactions[78]. Ellman and Katz et al. also suggested that host ant-tumor immunity is involved in the antitumor effect[79]. They reported that host antitumor immunity could be achieved even when the all ogeneic cells are already fully rejected and continuous tumor protection had been observed in 50% of rechallenged long-term survivors of allogeneic lymphocyte-infused animals [80]. These initial findings suggest that a GvH reaction is a prerequisite for a host-anti tumor activity to occur in thesetting of DLI, where RDICs are stimulated to elicit antitumor responses. In concordance, RDICs are presented as key players in the anticancer activity after allo-HSCT. Symons et al *.* reported that the transfer of CD8+ T-cell-depleted DLI graft into cyclophosphamide-treated A20 leukemia/lymphoma-bearing mice increased the survival directly through a GvH anti-tumorreaction of donor CD4+ T-cells and indirectly through stimulation of recipient CD8+ T-cell antitumor immunity [81].

Recipient derived antigen presenting cells (APCs) also play an important role during GvH reactions. In the early postallo-HSCT period, conditioning-induced tissue inflammation stimulates recipient APCs and they in turn prime alloreactive donor T-cells [82, 83]. Cross-presentation of recipient antigens by donor APCs may also occur after allo-HSCT. However, it still not clearly defined to what extent it occurs in human beings [83]. The role of recipient APCs in eliciting effective anticancer responses is very important and it is reflected in clinical studies reporting the outcome of DLI in advanced solid tumors. RDICs may have a principal effector role in the anticancer effect against renal cell carcinoma (RCC), as a significant tumor regression occurred despite a gradual decrease in donor chimerism[84]. This observation, reported by Harano et al., suggests that a temporary presence of donor cells is enough to create a GvH reaction and may provide inflammatory signals that facilitate the loss of tolerance of recipient CD8+ T-cells to the recipient’s tumor [84]. Similarly, Omazic et al. also showed a durable remission among patients with advanced refractory solid tumors in the presence of donor graft rejection [37].

As the preclinical and clinical evidences suggest that donor cells may only be needed in the initial induction phase of a GvT effect [37, 81], the research has focused on exploiting the potential of RDICs without increasing the risk of GvHD. Inmouse models of leukemia, Rubio et al. and De Somer et al. intentionally created graft rejection via “recipient leukocyte infusion” (RLI) [u2923]. A host-versus-graft (HvG) reaction created by RLI into mixed chimer as triggered a reaction of RLI-derived donor-reactive recipient T-cells and resulted in full donor graft rejection and an important antileukemic response without increasing the GvHD risk.

In summary, these findings support the initial reports suggesting that RDICs may act as key effectors in the anticancer effect after allo-HSCT. These results also strongly suggest that the effective anticancer responses mediated byRDICs are not solely through a GvHr eaction [u2924], but also a HvG reaction [u2925, u2926, u2927].

## 5. Chimeric antigen receptorT-cell (CAR-T) therapy for solid tumors 

Chimeric antigen receptor modified T-cell (CAR-T) therapy has achieved encouraging breakthroughs in the treatment of hematological malignancies. Nevertheless, this success has not yet been extrapolated to solid tumors [89]. Infact, the vast majority of cancers, in particular the more common solid cancers, including the breast, colon and lung, failed to respond significantly to CAR-T treatment. The suppression of T-cell function and inhibition of T-cell localization are some formidable barriers of solid cancers to adoptive cell transfer [90].

However, some promising results have also been reported in some early phase studies [91]. Phase 1 studies of GD2-specific CAR-T cells for neuroblastoma, CAR-T cells specifically targeting HER2, EGFR and IL-13 for glioblastoma multiforme, mesothelin-specific CAR-T cells for advanced malignant pleural mesothelioma or pancreatic cancer, CAR-T cells specific for epidermal growth factor receptor (EGFR) for advanced nonsmall-cell lung cancer and cholangiocarcinoma, CEA specific CAR-T cells for metastatic CRC have reported positive initial results [92–99].

Despite some promising results, the ultimate success of CAR-T therapies in solid tumors may require some adjustments and improvements. The combination of CAR-T cells with chemotherapy to treatmet as tatic tumors, local delivery of CAR-T cells,using CAR-T cells targeting two different antigens, combined therapy with CAR-T and immune check point inhibitors and finally the use of CAR-T as a strategy to prevent tumor recurrence and metastasis after curative resection are current questions to be further studied [89].

## 6. Tumor infiltrating T-cells in refractory solid tumors

The infiltration of the tumor tissue with T cells targeting tumor associated antigens has been shown to be associated with a favorable prognosis in several solid tumors. Upon this observation ongoing studies have been investigating the idea of extraction, ex vivo expansion with homeostatic cytokines and reinfusion into the patients as a novel treatment strategy [91]. Tumor infiltrating lymphocytes (TILs) were first reported by Rosenberg et al. in 1988 and they demonstrated the antimelanoma effects of IL-2 induced TILs [100]. The treatment with TILs and high-dose IL-2 has proven a 34% objective response rate [101–103]. TIL therapy has been reported to have lower response rates in patients progressed on anti-PD-1 therapy. However, TIL therapy remains an important treatment strategy in refractory malignant melanoma, as durable complete responses can still be induced after progression on anti-PD-1 [104].

Despite the demonstration of TILs in other solid tumors, their expansion and in vivo efficacy have not been a great success as in melanoma [u29a2] and some clinical trials in gastrointestinal, gynecological, head and neck, breast and lung cancers are currently ongoing [91].

TIL therapy in melanoma is an advanced therapy medicinal product and its clinical implementation is challenging. Thus, it has not been widely recognized. It has been availablein the Europe since 2011 as an experimental therapy. Reimbursement procedures and organization of knowledge transfer could improve clinical translation of TIL therapy [107].

## 7. Summary

Current evidence suggests the presence of graft-versus-cancer effect in various solid tumors. Allo-HSCT with RIC may provide some degree of response in some refractory metastatic solid tumors, such as renal, ovarian, breast and even colon cancers. Lower toxicity profile and lower nonrelapse mortality rate make RIC regimens a plausible treatment modality. To date, the results of this treatment modality in refractory solid tumors are associated with lower CR and PR rates with few long-term survivors, which is similar to CAR-T Cell experiences in refractory solid tumors. Current literature data imply that mechanisms of GVT effect and interaction of T-cells and their subsets with main mediators may be highly different in solid tumors compared to hematologic malignancies. Therefore, further studies are needed shedding light upon these mechanisms in order to exploit this valuable effect in refractory solid tumors. 

Despite its great potential, the use of ATCT for cancer control yet has a marginal role in the management of patients with solid tumors. However, it has recently come into attraction in melanoma treatment [36]. Indeed, the extensive infrastructure needed for exploiting ATCT effects still restrict its use to academic centers with specific programs in the field. It should be emphasized that the major obstacle for a wider application of ATCT to treat human cancer is the personalized nature of the approach [36].

Although donor T-cells are accepted as the main mediators of the anticancer effect following allo-HSCT, recent findings also point out a key role for RDICs. Recent experimental studies appointed RLI as an important tool to reinforce anticancer effects after allo-HSCT by exploiting RDICs, both in leukemia and solid tumor models with an advantage of lower rates of GvHD. These results supporting the contribution of RDICs in the anticancer effect of allo-HSCT are mainly observed in murine models, and the experience in human is limited. Future clinical trials may explore the emerging role and anticancer effects of RDICs in patients receiving allo-HSCT.

Further studies and experience are warranted regarding the use of haploidentical HSCT, CAR-T cell therapies, posttransplant immunomodulatory agents and tumor infiltrating T-cells in patients with refractory solid tumors [89, 90, u29a5]. Future studies should include patients with better performance status and chemotherapy responsive disease before transplant in order to obtain the maximal benefit from GvT effect in solid tumors. Well-designed trials are needed for a clear-cut understanding of the interactions of donor T-cells and their subsets, mechanisms of GvT effects, which possibly use different mechanisms in solid tumors and hematologic malignancies, in order to optimize the efficacy of such treatment modalities in patients with refractory solid tumors.
